# Clinical significance of altered *nm23-H1, EGFR, RB and p53 *expression in bilharzial bladder cancer

**DOI:** 10.1186/1471-2407-9-32

**Published:** 2009-01-26

**Authors:** Hussein M Khaled, Abeer A Bahnassy, Amira A Raafat, Abdel-Rahman N Zekri, Maha S Madboul, Nadia M Mokhtar

**Affiliations:** 1 Medical OncologyDepartment, National Cancer Institute, Cairo University, Cairo, Egypt; 2 Pathology Department, National Cancer Institute, Cairo University, Cairo, Egypt; 3 Cancer Biology Department, National Cancer Institute, Cairo University, Cairo, Egypt; 4 Clinical Pathology Department, National Cancer Institute, Cairo University, Cairo, Egypt

## Abstract

**Background:**

Clinical characterization of bladder carcinomas is still inadequate using the standard clinico-pathological prognostic markers. We assessed the correlation between *nm23-H1*, *Rb, EGFR *and *p53 *in relation to the clinical outcome of patients with muscle invasive bilharzial bladder cancer (MI-BBC).

**Methods:**

*nm23-H1*, *Rb, EGFR and p53 *expression was assessed in 59 MI-BBC patients using immunohistochemistry and reverse transcription (RT-PCR) and was correlated to the standard clinico-pathological prognostic factors, patient's outcome and the overall survival (OS) rate.

**Results:**

Overexpression of *EGFR *and *p53 *proteins was detected in 66.1% and 35.6%; respectively. Loss of *nm23-H1*and *Rb *proteins was detected in 42.4% and 57.6%; respectively. Increased *EGFR and *loss of *nm23-H1 *RNA were detected in 61.5% and 36.5%; respectively. There was a statistically significant correlation between *p53 *and *EGFR *overexpression (*p *< 0.0001), *nm23 *loss (protein and RNA), lymph node status (*p *< 0.0001); between the incidence of local recurrence and *EGFR *RNA overexpression (p= 0.003) as well as between the incidence of metastasis and altered *Rb *expression (*p *= 0.026), *p53 *overexpression (*p *< 0.0001) and mutation (*p *= 0.04). Advanced disease stage correlated significantly with increased *EGFR *(protein and RNA) (*p *= 0.003 & 0.01), reduced *nm23-H1 *RNA (*p *= 0.02), altered *Rb *(*p *= 0.023), and *p53 *overexpression (*p *= 0.004). OS rates correlated significantly, in univariate analysis, with *p53 *overexpression (*p *= 0.011), increased *EGFR *(protein and RNA, *p *= 0.034&0.031), *nm23-H1 RNA *loss (*p *= 0.021) and aberrations of ≥ 2 genes. However, multivariate analysis showed that only high *EGFR *overexpression, metastatic recurrence, high tumor grade and the combination of ≥ 2 affected markers were independent prognostic factors.

**Conclusion:**

*nm23-H1, EGFR *and *p53 *could be used as prognostic biomarkers in MI-BBC patients. In addition to the standard pathological prognostic factors, a combination of these markers (≥ 2) has synergistic effects in stratifying patients into variable risk groups. The higher is the number of altered biomarkers, the higher will be the risk of disease progression and death.

## Background

In Egypt, Schistosoma-associated bladder cancer represents the commonest malignancy in all diagnosed cancer cases according to the registry of the National Cancer Institute, Cairo [1]. To date, several studies have attempted to identify the spectrum of genetic changes that occurs during urothelial transformation of bilharzial bladder cancer (BBC) and to elucidate in detail the natural history of tumors with different clinical outcome. A wealth of information about the molecular pathogenesis of BBC has emerged, including cytogenetic and molecular genetic analysis via comparative studies on schistosoma- and non-schistosoma- associated bladder cancers which demonstrate different clinicopathologic features, pathogenetic mechanisms and a unique genetic make-up. However, the clinical significance of these defects either singular or in combination, is still not clear [2-4].

Some of these studies documented a significant reduction in disease free survival (DFS) for *p53 *positive tumors in BBC and transitional cell carcinoma (TCC) of the western countries [3,5] Similarly, absence of *Rb *protein was found more frequently in tumors with high grade and stage and was clearly associated with poor clinical outcome [6-9] However, the examination of data for single markers is not sufficient to direct clinical decisions for individual patients [4].

The *nm23-H1 *gene *(NME1)*, localized on chromosome 17q21.3 was first isolated as a metastasis suppressor gene by differential screening of cDNA library from high and low metastatic clones of a murine melanoma cell line [10]. The clinical relevance of the *nm23-H1 *as a metastasis suppressor for human cancers including bladder is still controversial. Some studies demonstrated that *nm23-H1 *is inversely correlated with tumor staging, histological differentiation and clinical outcome [11,12], others showed a positive relationship to histological grading and muscle invasion [13].

Epidermal growth factor receptor (*EGFR*) is a member of the tyrosine kinase receptor family, a group of receptors which are all encoded by the c-*erb*B oncogenes. It is situated on chromosome 7 and is a 170 kDa protein that consists of three distinct structural parts. It has been proved that epidermal growth factor signaling plays a pivotal role in tumorigenesis and disease progression. Overexpression of *EGFR *leads to uncontrolled cell proliferation, increased angiogenesis and reduced apoptosis, processes necessary for continuing malignant growth [14]. The role of *EGFR *in urothelial tumors was supported by the observation that 40%–60% of human bladder tumors overexpress *EGFR *mRNA and protein [15]. In addition, some studies showed a strong correlation between *EGFR *positivity and high grade, late stage, tumor progression and poor clinical outcome in the classic TCC of the bladder [16-18].

The present study was conducted to assess the prognostic impact of altered expression of *nm23-H1, EGFR*,*Rb *and *p53 *gene status either singular or in combination in Egyptian cases of muscle invasive-BBC (MI-BBC). Aberrations involving these markers will be correlated to the standard prognostic factors of bladder cancer, patients' response to treatment, and overall survival (OS).

## Methods

### Patients and tissue samples

The study included 59 cystectomy specimens obtained from operable cases of MI-BBC that were diagnosed and treated at the National Cancer Institute (NCI), Cairo University during the years 1996–1999. From each case, a section was obtained from the paraffin block of the tumor, stained with hematoxylin and eosin and examined under microscope by two independent pathologists (MNM & BAA) to confirm the diagnosis, typing and grading of the tumor as well as to calculate tumor: normal ratio. Only cases in which the neoplastic cells constituted ≥ 75% were included in the study. Also included are 15 normal urothelial tissue samples which were used as a control. These were obtained from morphologically normal areas as far away as possible from tumors not exceeding 1.5 cm in diameter and were confirmed to be normal by microscopic examination of hematoxylin and eosin- stained slides.

None of the cases have received neo-adjuvant or adjuvant therapy prior to surgery. All Clinico-pathological data of the patients including patients' age, sex, tumor type, grade and stage, lymph node status, local recurrence, distant metastasis and overall survival (OS) rates were retrospectively collected from the pathology reports and clinical files of the patients. All studied tumors were graded according to The World Health Organization (WHO) classification and staged according to the TNM pathological staging system [19]. Patients were followed up for local recurrence (LR), distant metastasis (DM) and overall survival (OS). The follow-up period ranged from 1–94 months with a median of 19 months. The ethical committee of the NCI approved the protocol which was in accordance with the ethical guidelines of the 1975 Declaration of Helsinki.

From each case (tumor and normal), 4 um thick sections (4) were cut onto positive-charged slides for immunohistochemistry and three 12 um thick sections were cut into Eppendorf tubes for RNA extraction.

### Immunohistochemistry

Sections were deparaffinized in xylene, hydrated through a series of graded alcohol, washed in distilled water and 0.01 PBS (pH 7.4), immersed in 10 mmol/L citrate buffer (pH 6.0) and put in a microwave for 5 min at 60°C for antigen retrieval. Then they were placed in methanol containing 3% H_2_O_2 _for 30 min at 4°C to block endogenous peroxidase activity and incubated with rabbit serum for 10 min to block non-specific antibody binding sites. The primary antibody was applied at a working concentration and incubated for 2 hours at 4°C. The monoclonal antibodies used are: antihuman *Rb1 *(*Rb1*, 1:80, DAKO), anti-human *nm23-H1 *(1:50; Santa Cruz, CA, USA) and anti-human *EGFR *(clone H11, 1:50, DAKO). The secondary antibody and the avidin-biotin complex (ABC) were applied to slides. Diaminobenzidine (DAB) was used as a chromogen and sections were counterstained with Mayer's hematoxylin. Negative controls were obtained by replacing the primary antibody by non-immunized rabbit or mouse serum [20].

A distinct brown nuclear staining was scored positive for *Rb. *The staining pattern of *nm23-H1 *was cytoplasmic and *EGFR *was membranocytoplasmic. Altered expression of *Rb *was defined as absence of nuclear staining in all sections examined [20]. Evaluation of *nm23-H1 *was performed as previously described [21]. Cases were scored negative with < 5% positive cells, +; with ≥ 5%–<25%, ++; with ≥ 25%–<70%, and +++; with ≥ 70% immunostaining of tumor cells. The cutoff point for *EGFR *was 10% and the intensity of staining was scored as weak; 10–<25%, moderate; 25–<50% and marked >50% **(15). **At least 500 cells were counted at 200× magnification.

### RNA extraction and PCR amplification

Total RNA was extracted from paraffin-embedded tissue sections according to the described methodology of pure script RNA isolation kit, (Gentra, USA). The extracted total RNA was assessed for degradation, purity and DNA contamination by spectrophotometry and electrophoresis in 1.0% ethidium bromide-stained agarose gel. Reverse transcription (RT) of the isolated total RNA was done using the Superscript One-Step RT-PCR Kit with Platinum *Taq *(Life Technologies, Inc.) in a 50 ul reaction volume containing 1 μl of Superscript II RT enzyme, 2.5 ul 10× RT-buffer [250 mM Tris-HCl pH8.3, 375 mM KCl, 15 mM MgCl_2_], 0.25 μl of 100 mM dithiothreitol, 1 μl of 25 ng from random primer, 1.5 μl of 10 mM deoxynucleotide triphosphates, 0.5 μl RNAsin (Promega, USA.), and 1.0 μg of the extracted total RNA (each sample as well as the controls). Samples were then incubated at 50°C for 60 min, followed by 4°C until the PCR amplification reaction. PCR amplification was done as previously described by Raynor et al. [22] and Ayabe et al. [23]. The primers used and the PCR conditions for each marker gene are illustrated in table 1. Amplification of *B-actin *gene (621-bp fragment) was performed to test for the presence of artifacts and to asses the quality of RNA. A water control tube containing all reagents except c-DNA was also included in each batch of PCR assays to monitor contamination of genomic DNA in the PCR reagents. Negative RT-PCR control was used against each sample.

**Table 1:** Primer sequences, expected product size in nucleotides (nt) and PCR conditions for each of the studied marker genes.

*Primer*	*Sequences*	*Fragment size*	*PCR conditions*
*nm23-H1*	5'-CGCAGTTCAAACCTAAGCAGCAGCTGG-3' 5'-AGATCCAGTTCTGAGCACAGCTCG-3'	483 bp	95°C for 5 m followed by: 95°C (30s), 55°C(30s), 72°C(30s) for 32 cycles and 72°C (7 m)

*EGFR*	5'-TGTGAGGTGGTCCTTGGGAATTTGG-3' 5'-TGCTGACTATGTCCCGCCACTGGA-3'	322 bp	95°C for 5 min followed by: 94°C (1 m), 66°C (1 m), 72°C (2 m) for 35 cycles and 72°C (7 m)

*β-actin*	5'-ATCTGGCACCACACCTTCTACAATGAGGCTGCG-3' 5'-CGTCATACTCCTGCTTGCTGATCCACATCTGC-3'	838 bp	95°C for 5 min followed by: 94°C (45s), 60°C (45s), 72°C (2 m) for 35 cycles *and *72°C (7 m)

### Quantization of the studied genes

Fifteen microliters of each PCR product were separated by electrophoresis through a 2.0% ethidium bromide-stained agarose gel and visualized with ultraviolet light. Gels were video-photographed the bands were scanned as digital peaks, areas of the peaks were then calculated in arbitrary units with a digital imaging system (Photo-documentation system, model IS-1000; Alpha Innotech Co., San Leandro, CA, USA). To evaluate the relative expression levels of target genes in the RT-PCR, the expression value of the normal pooled urothelial tissues (15 samples) was used as a normalizing factor and a relative value was calculated for each target gene amplified in the reaction. Loss of expression in any of the studied genes was considered if there was a complete absence or more than a 75% decrease in the intensity of the desired band, in comparison to the band of normal pooled liver tissue [24]. Samples were assayed in batches that included both cases and controls. The absence of bands was verified by repeating the RT-PCR twice at different days and consistent presence of *B-actin *gene amplification.

### Statistical analysis

Numerical data were expressed as mean ± standard deviation (SD), median, minimum and maximum. Qualitative data were expressed as frequency and percentage. Chi square test (Fisher's exact test) was used to examine the relation between qualitative variables. For quantitative data, comparison between two groups was done using Mann-Whitney test. Survival analysis was done using Kaplan-Meier method. The OS duration of the patients was calculated from the date of diagnosis to the date of death. Comparison between two survival curves was done using log-rank test. Probability (p-value) ≤ 0.05 was considered significant. The multivariate analysis (MVA) was done using Cox proportional hazard model. Factors entered in the MVA were significant or of borderline significance in univariate analysis.

## Results

In the present study, 59 well-characterized cases of MI-BBC were assessed for the expression level and the prognostic value of *pRb, nm23, and EGFR *proteins as well as RNA using immunohistochemistry and RT-PCR. The patients' age ranged from 30–76 years with a median of 50 years, the male: female ratio was 2.47:1.0. Twenty one out of the 54 patients for whom data regarding metastatic recurrence was available (38.8%) showed evidence of recurrence either as local (13), distant (8) or both (5). The median OS of the patients was 27 ± 6.6 months with the cumulative OS rate of 56% at 18 months. The presenting clinicopathological features and survival status of the whole group of patients are shown in table 2.

**Table 2:** Clinicopathological features of the 59 patients studied

Parameter	No of Cases (%)
**Sex:**	
Male	42 (71.2)
Female	17 (28.8)

**Pathological Subtype:**	
SCC	27 (45.8)
TCC	27 (45.8)
Adeno	5 (8.5)

**Grade:**	
I	5 (8.5)
II	39 (66.1)
III	15 (25.4)

**Pathological Stage:**	
P2	3 (5.08)
P3a	24 (40.06)
P3b	26 (44.06)
P4	6 (10.2)

**Lymph Node Involvement:**	
Positive	10 (16.9)
Negative	49 (83.1)

**Local recurrence:**	
No	41 (69.5)
Yes	13 (30.5)*

**Metastasis:**	
Negative	46 (78)
Positive	8 (22.03)*

**Outcome:**	
Dead	32 (54.2)
Alive	27 (45.8)

* Data regarding recurrence and metastasis was available for 54 cases only

### Expression of the studied markers

A positive cytoplasmic immunostaining for *nm23-H1 *as well as a positive nuclear immunostaining for *Rb *were detected in the 15 normal urothelium samples included in the study as a control. The *nm23-H1 *positive cells were restricted to the superficial layer only whereas *Rb *positivity was recognized in all layers. On the other hand, no immunorectivity for *p53 *or *EGFR *proteins was detected in the control samples.

**Figure 1:**
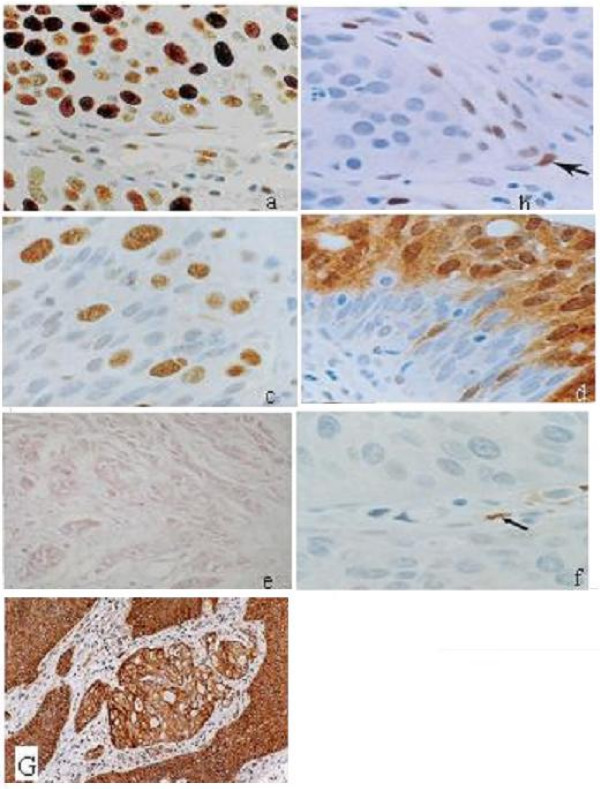
**Protein expression of the studied markers in cases of muscle invasive bilharzial bladder cancer**: Cases of BBC showing (a) positive immunostaining for *p53*, (b) negative immunostainning for *Rb*, (c) positive heterogeneous immunostaining for *Rb *(d) normal *nm23-H1*, (e) reduced *nm23-H1 *expression, (f) loss of *nm23-H1 *expression in neoplastic cells with positive expression in endothelial cells (arrow-positive control), (g) *EGFR *overexpression.

**Figure 2:**
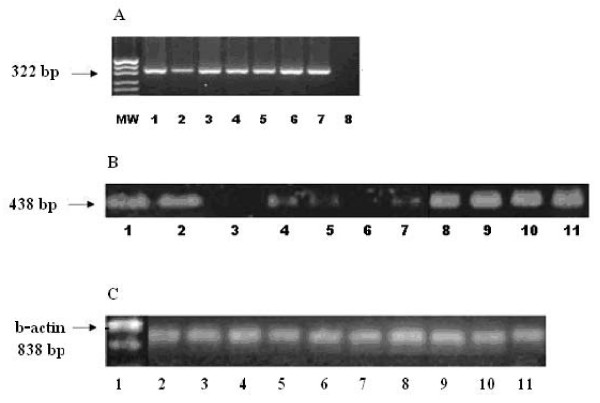
**RNA expression of *EGFR*, *nm23-H1 *and *β actin *in cases of muscle invasive bilharzial bladder cancer**: (a) Expression of *EGFR *by RT-PCR: first lane; molecular weight marker, lanes 1–7: cases of BBC showing *EGFR *expression, lane 8: a negative control, (b) *nm23-H1 *RNA expression by RT-PCR: Lanes 1, 2, 8–11: normal expression of *nm23-H1*, lanes 3 and 6: loss of expression of *nm23-H1*, and lanes 4, 5, and 7: reduced expression of *nm23-H1*, and (c) Lanes 1–11: Expression of *β actin *in cases of BBC.

Loss of *nm23 *protein was detected in 25/59 cases (42.4%) whereas, positive cytoplasmic immunostaining was evident in 34/59 (57.6%) cases; 8 of them were minimally positive and 10 were markedly positive (Figure 1d, e, and 1f). RNA was successfully extracted from 52 out of the 59 cases included in the study whereas in the remaining 7 cases, either no RNA or a degraded RNA was present. This is explained by the difficulty in obtaining RNA from paraffin-embedded tissues even with the use of special kits available. Loss of *nm23-H1 *RNA was detected in 19/52 (36.5%) of the cases (Figure 2b). The concordance between the loss of *nm23-H1 *protein and *nm23-H1 *RNA expression was 84.6%.

A positive membranous immunostaining for *EGFR *protein was detected in 39/59 (66.1%) cases; 20 of them were markedly positive, 9 were moderately positive and 10 were minimally positive (Figure 1g). Increased *EGFR *RNA was detected in 32 out of the 52 (61.5%) evaluable cases (Figure 2a). The concordance between the increased expression of *EGFR *protein and RNA was 98.1%.

Altered *pRb *expression, defined as negative nuclear staining, was detected in 34/59 (57.6%) cases whereas 25 cases (42.3%) were normal with either homogeneous or heterogeneous nuclear immunostaining (Figures 1b, and 1c).

We have previously assessed the same series of BBC for *p53 *protein expression and gene mutations [3]. Positive nuclear immunostaining for *p53 *was detected in 21 out of the 59 cases included in the present study (35.6%); of which 6 were minimally positive and 15 were markedly positive (Figure 1a). Mutations of the *p53 *gene (exons 5 through 10) were detected in 8 cases only (13.6%) with no mutational hotspots. Four cases showed a single mutational event and 4 revealed several mutations in more than one exon.

Statistical analysis revealed no significant relation between the expression level of the studied proteins except for the highly significant relation between *p53 *and *EGFR *overexpression where 20 out of the 21 (95.2%) *p53 *positive cases revealed *EGFR *overexpression while a single case only (4.8%) showed normal *EGFR *protein expression (p < 0.0001).

### Correlation between the studied markers and standard prognostic factors

As shown in table 3, there was a significant correlation between positive lymph node status and reduced *nm23-H1 *(protein &RNA) (*p *< 0.0001) and *p53 *overexpression (*p *= 0.02). Also, there was a significant correlation between advanced disease stage (p2& p3a *vs. *p3b& p4) and reduced *nm23-H1 *RNA expression (*p *= 0.02), increased *EGFR *(protein & RNA) (*p *= 0.003 & 0.01 respectively), altered *Rb *expression (*p *= 0.023) and *p53 *overexpression (*p *= 0.004). Similarly, there was a significant correlation between the incidence of recurrence and increased *EGFR *RNA (p = 0.003) and a marginally significant correlation with *EGFR *protein overexpression (*p *= 0.064) as well as between metastasis and *p53 *overexpression (*p *< 0.0001), *p53 *mutation (*p *= 0.04) and altered *Rb *expression (*p *= 0.026).

**Table 3:** The correlation between aberrant expression level of the studied markers and the clinicopathological features of the patients

Parameter	*nm23-H1* IHC(25)	*nm23-H1* RNA (19)	*EGFR* IHC(39)	*EGFR* RNA (32)	*Rb* IHC(34)	***P*53** IHC(21)
***Age(mean ± SD)***	50.2 ± 11	54.o ± 8.6	51.7 ± 11	48.5 ± 10.2	49.5 ± 11.4	53.9 ± 11.3
*p value*	0.41	0.63	0.73	0. 41	0.144	0.175

***Sex***						
*Male (42)*	19	13	28	25	22	17
*Female (17)*	6	4	11	7	12	4
*p value*	0.48	0.42	0.88	0.62	0.2	0.22

***Pathology:***						
*SCC*	14	9	16	9	16	7
*TCC*	10	8	20	20	16	12
*AC*	1	2	3	3	2	2
*P value*	0.536	0.661	0.21	0.365	0.85	0.36

***Grade:***						
*I*	1	2	3	3	4	0
*II*	17	15	25	20	22	16
*III*	7	2	11	9	8	5
*P value*	0.916	0.713	1.0	0.785	0.71	0.224

***Stage:***						
*p3a*	15	4	26	17	25	17
*p3b*	9	10	7	9	6	2
*p4*	1	5	6	6	3	2
*P value*	0.13	***0.02***	***0.003***	***0.01***	***0.023***	***0.004***

***Lymp nodes:***						
*Positive*	9	6	8	4	4	6
*Negative*	16	13	31	28	30	15
*P value*	***<0.0001***	***<0.0001***	*0.31*	0.431	0.216	***0.02***

***Recurrence:***						
*Negative*	16	15	26	24	23	13
*Positive*	9	4	13	8	11	8
*P value*	0.43	0.51	***0.06***	***0.003***	0.72	0.347

***Metastasis:***						
*Negative*	20	15	31	23	30	13
*Positiv****e***	5	4	8	9	4	8
***P value***	0.75	0.51	0.78	0.511	***0.026***	***<0.0001***

SCC = Squamous Cell Carcinoma; TCC = Transitional Cell Carcinoma; Adeno = Adenocarcinoma

### Survival analysis

**Figure 3:**
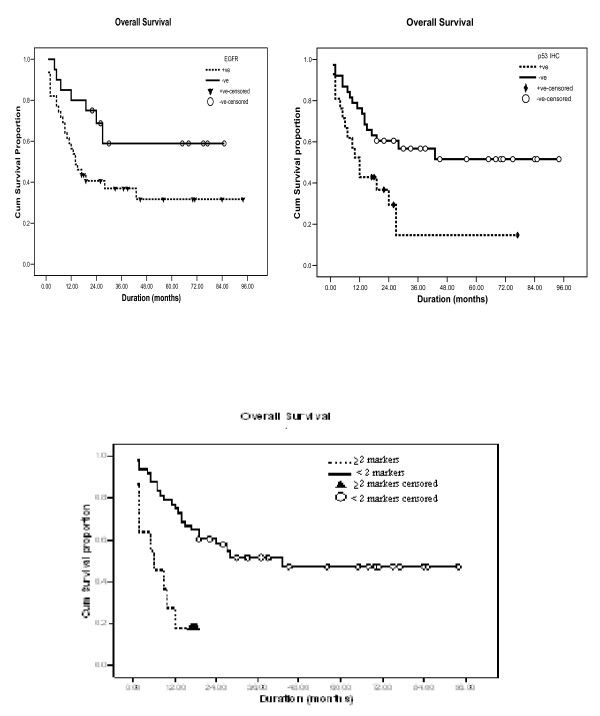
**Kaplan-Meier analysis for cases of BBC**. Overall survival is significantly lower in patients with: (a) increased *EGFR *expression, (b) *p53 *overexpression, (c) combined (*nm23 *-ve and *EGFR *+ve), (d) combined (*nm23 *-ve and *p53 *+ve),

On univariate analysis there was a statistically significant correlation between reduced OS rates and tumor type (SCC) (*p *= 0.043), high tumor grade (*p *= 0.008), local recurrence (*p *= 0.0001) and distant metastasis (*p *= 0.0001). A borderline significance was reported between advanced disease stage (3b+4 ***vs ***2+3a) and reduced OS. Similarly reduced OS rates was significantly associated with loss of *nm23-H1 *RNA (*p *= 0.021), increased *EGFR *protein (Figure 3a) and *EGFR *RNA expression (*p *= 0.034 &*p *= 0.025) and *p53 *overexpression (*p *= 0.011) (Figure 3b). The cumulative survival for cases with normal *nm23-H1 *RNA expression was 64.8% whereas it was 35.5% for cases with reduced/lost *nm23-H1 *RNA. Likewise, the cumulative survival for *EGFR *positive patients was 41.0% compared to 69.0% for *EGFR *negative patients and 29% for patients with *p53 *overexpression *vs *60% for patients with no overexpression. On the other hand, the cumulative survival for *nm23-H1 *protein overexpression, altered *Rb and p53 *mutation did not differ significantly between negative and positive groups (*p *values 0.345, 0.287 and 0.539; respectively). On multivariate analysis, only increased *EGFR *expression (protein or RNA), the presence of metastasis and/or recurrence, a high tumor grade (GII &GIII) and a combination of aberrant markers (≥ 2) were significantly associated with reduced OS rates (*p *= 0.022&0.025, *p *= 0.000, *p *= 0.001, *p *= 0.001). On the other hand, *p53 *overexpression and advanced tumor stage (3b &4 vs 2&3a) showed a borderline significance (table 4)

**Table 4:** Overall survival in relation to standard clinicopathological prognostic factors and the studied markers

		Univariate analysis			Multivariate analysis		
Predictive variables	CS	HR	CI	p	HR	CI	p
**Age**							
**<**47	68%	1	0.99, 1.02	0.880			
≥ 47 *	66%						

**Sex:**							
Male (42)	59%	1.1	0.85, 1.04	0.652			
Female (17)	64%						

**Tumor type**							
SCC (27)	60%	1.212,	0.538, 2.734	0.643			
TCC (27)	65%						
Adenocarcinoma (5)	60%						

**Tumor grade**							
I (5)	66%	1.96	1.66, 21.331	**<0.000**	4.350	1.786, 10.591	**0.001**
II (39)	32%						
III (15)	11%						

**Lymph Node Involvement:**		1.099	0.423, 2.859	0.846			
Positive (10)	49%						
Negative (49)	55%						

**Metastatic recurrence:**							
No (33)	58%	14.149	5.117, 39.123	**0.000**	14.903	5.315, 41.785	**0.000**
Yes (21)	25%						

**Pathological Stage:**							
p2 & pp3a (27)	49%	1.341	0.575, 3.127	0.498			
p3b &p4 (32)	35%						

**Nm23 (IHC***)*							
Positive (34)	66.5%	1.62	0.535, 2.244	0.345			
Negative (25)	59.6%						

**nm23(RNA***)*							
Positive (33)	64.8%	1.34	1.20, 1.50	**0.021**	0.695	0.586, 1.975	0.444
Negative (19)	35.5%						

**EGFR (IHC)**							
Positive (39)	41%	1.61	1.04, 2.29	**0.034**	3.576	1.206, 10.602	**0.022**
Negative (20)	69%						

**EGFR (RNA)**							
Positive (32)	39%	1.69	1.16, 2.74	**0.031**	3.911	1.148, 8.511	**0.025**
Negative (20)	69%						

**Rb**							
Positive (25)	54%	1.1	0.27, 1.76	0.287			
Negative (34)	49%						

**p53 (IHC)**							
Positive (21)	29%	3.9	2.281, 12.42	**0.011**	2.512	0.948, 6.662	0.64
Negative (38)	60%						

**P53 (mutations)**							
Positive (8)				0.539			
Negative (51)							

**Number of abnormal genes**		1.70	1.20, 5.90	**0.022**	7.331	2.696, 12.940	**0.001**
**<**2							
**≥ **2							

* The median age for the studied patients

** Data regarding metastatic recurrence was available for 54 cases only

The OS rates were also assessed in relation to combined genetic aberrations involving more than one gene (Table 5). Cumulative survival for altered expression of *nm23-H1 *and *p53 *(combined) was 11.0% as compared to 57.0% for other combinations (*p *= 0.0004), while that for altered expression of *nm23-H1 *and *EGFR *(combined) was 28.0% as compared to 60.0% for other combinations (*p *= 0.029). The cumulative survival for altered expression of *EGFR *and *p53 *(combined) was 18.0% as compared to 58% for other combinations (*p *= 0.0002) (Figure 3c–e). However, combined *nm23-H1 *loss and altered *Rb*, altered *Rb *and *p53 *overexpression as well as altered *Rb *and *EGFR *overexpression did not show an impact on survival (*p *= 0.438, 0.174, and 0.875; respectively). A statistically significant correlation was also present between OS and aberrations involving the 4 studied markers (p = 0.0001).

**Table 5:** Correlation between survival and combined marker expression

	No.	Cumulative survival (%)	Median ± SE	95% CI	p. value
***Nm23*(-)&*p53*(+)**	9	11	6 ± 1.49	3.08 – 8.92	
Other combinations	50	57	43 ± -	-	***0.0004***

***Nm23*(-)&*EGFR*(+)**	18	28	10 ± 3.18	3.76 – 16.24	
Other combinations	41	60	43 ± -	-	***0.029***

***EGFR*(+)&*p53*(+)**	11	18	6 ± 3.85	0 – 13.55	
Other combinations	48	58	43 ± -	-	***0.0002***

***Nm23*(-), *p53*(+),*EGFR*(+)**	6	15	5.8 ± 3.85	0.1–13.55	***0.0001***
Other combinations	53	59	43 ± -	-	

***Nm23*(-), *p53*(+),*EGFR*(+)&*b (-)***	7	9	4 ± 5.62	1.45 – 20.21	***<0.0001***
Other combinations	52	58	50 ± -	-	

## Discussion and Conclusion

Molecular studies of bladder tumors have identified several genetic alterations including self insufficiency in growth signals, evasion of apoptosis, sustained angiogenesis, acquisition of indefinite replication potential and metastasis. However, only few of these have proven to be potentially positive clinical targets in the prognosis and therapy of bladder cancer [25].

The present study addresses, for the first time, the role of *nm23-H1 *and *EGFR *aberrations (at the RNA and protein levels) in Egyptian cases of muscle invasive BBC in relation to *RB*, and *p53 *and illustrates the clinical significance of these aberrations either singular or in combination.

Reduced expression of *nm23-H1 *protein and *nm23-H1 *RNA was significantly associated with positive lymph nodes, advanced disease stage (p3a *vs *p3b&p4) and reduced overall survival (OS) rates. Taken together, it could be suggested that, *nm23-H1 *may be a suppressor for BBC progression like in some tumors of other organs e.g. breast, liver, stomach, ovary, and colon [21,26-28].

Data regarding *nm23-H1 *in TCC of the bladder in western countries are contradictory. Our results are consistent with several studies [12,29-31] which showed a significantly inverse correlation between reduced *nm23-H1 *expression and disease staging, histopathologic differentiation, tumor size, high risk of metastasis and reduced OS. However, some reports showed a positive correlation between *nm23-H1 *expression and tumor grading, muscle invasion or proliferating cell nuclear antigen expression, implying a positive growth regulatory role for *nm23-H1 *in bladder carcinogenesis [13,31,32].

This discordance in the reported results may be partly explained by differential specificity of the antibodies applied in the earlier reports. In our work as well as that of Chow and co workers [29], a monoclonal antibody reactive to purified *nm23-H1 *of human origin was used, whereas polyclonal *nm23-H1 *antibody [12,31] and monoclonal anti-NDP (nucleoside diphosphate) kinase antibody [32] were used in some prior studies. With respect to NDP kinase, the enzyme activity has been proved to be unrelated to tumor suppression [33]. The high sensitivity and specificity of the monoclonal antibody used in the present study was confirmed by the 100% concordance reported between the protein and RNA expression levels.

Our results regarding the frequency of increased *EGFR *expression (62.3% and 66.1%; protein and RNA respectively) is within the universally reported range (60–75%) in non- bilharzial bladder cancer cases [34,35]. In BBC, the role of *EGFR *was addressed in two studies only. In the first study [20], *EGFR *overexpression was detected in 67% of the cases with no significant relation reported with any of the clinicopathological prognostic factors or survival. In the second study **(36) **the authors reported *EGFR *loss in high grade SCC but not TCC.

We also found that increased expression of *EGFR *protein and/or RNA were significantly associated with advanced disease stage, increased incidence of local recurrence and reduced OS. The significant association reported in the current study between increased *EGFR *expression and high tumor stage is in agreement with several previous studies on TCC of the western countries [34,36]. However, Popove et al [16] demonstrated that *EGFR *expression had no additional prognostic value over clinical stage, grade or cell proliferation. The study of Memon et al. [37], provided an explanation for this controversial data since they found that in some cases *EGFR *expression alone shows no correlation with survival, yet a high expression of *EGFR *together with increased *Her3 *and *Her4 *correlate with a better survival compared to increased *EGFR *together with decreased *Her3 *and *Her4*. This denotes the existence of a cooperative/synergistic effect between *EGFR *and other *ERB *family members mainly *Her3/4*.

In a recent study, Calquhom et al. [38] found a trend towards improved bladder cancer-specific survival in *EGFR *negative patients. Moreover, a positive response to radiotherapy significantly correlated with a negative *EGFR *status in the studied patients. Similarly Munk et al. [39] illustrated that *EGFR *activation induces cell survival in RT4 and T24 bladder cancer cell lines treated with DNA damaging chemotherapeutic agents. Therefore, they concluded that combined treatment with these drugs and *EGFR *inhibitors might improve the efficacy of bladder cancer treatment.

Numerous studies have claimed that inactivation of *p53 *and *Rb *plays a pivotal role in the pathogenesis of BBC and none bilharzial bladder cancer [5,9,40-42]. Our results are similar to those of Loachim et al. [43] who found altered expression of *Rb *and *p53 *proteins in 55.2% and 33.3% of their studied cases. On the other hand most previous studies reported lower rates, varying from 16–39% for *Rb *expression. These studies included either superficial bladder tumors only [41] or a mixture of superficial and muscle invasive tumors [5,42], whereas the present study includs muscle invasive tumors only. The higher frequency found in BBC cases could also be explained by the high frequency of HPV infection (46%–100%) reported by several investigators in Egyptian cases of BBC [3,44,45].

The clinical significance of *Rb *loss is still conflicting. Our results are consistent with some previous studies [42,46] which showed that *Rb *negative tumors had a more aggressive biologic behavior with decreased patients' survival, and more liability to tumor progression than positive ones. Thus loss of *Rb *expression can be used as a prognostic factor in bladder cancer. However, some other studies found no correlation between altered *Rb *expression and any of the known prognostic variables [5,47].

Our results regarding *p53 *mutation and/or protein overexpression in BBC and its prognostic value are comparable to previous reports on BBC and some non bilharzial bladder cancer [3,5,7,44]. However, our frequency is lower than Helal et al [48] who detected *p53 *overexpression in 52% of in non bilharzial bladder cancer cases and in and 56.3% of BBC cases. The difference between our results and those of Helal et al [48] could be attributed to the presence of associated HPV infection in our studied cases which lead to degradation of the *p53 *protein (**3**) whereas Helal et al. were not able to find HPV-DNA by *in situ *hybridization in their studied cases. The prognostic value of *p53 *which was reported in the current study has been previously reported in several studies on bladder cancer [3,42,46,49].

An interesting and novel finding in the present study is the correlation between aberrant expression of the studied markers and the OS rates and the use of marker combination for the assessment of the clinical outcome of patients. According to our data patients were categorized into two groups with a statistically significant difference between these groups with respect to OS rates: patients with aberrations in a single gene and those with more than one abnormal gene (Tables 4&5). This denotes the significance of using a panel of genes for predicting patient's outcome rather than using a single gene. Similar results have been reported by Shariat et al. [42] who found in their study of superficial cases of TCC of the bladder that a combination of biomarkers (*p53, Rb, p21 and p27*) are useful in stratifying patients into different risk groups for disease recurrence and progression.

We conclude that aberrant expression of *nm23-H1, EGFR, p53 and RB *is a frequent event in BBC. A high *EGFR *and *p53 *expression could be added to the existing clinico-pathologic prognostic factors. Moreover, a combination of these markers or some of them has synergistic effects in stratifying patients into different risk groups: those with more than one abnormal gene have a higher risk of disease progression and a reduced OS whereas those with a single abnormal gene have a better clinical outcome and OS rates.

## Competing interests

The authors declare that they have no competing interests.

## Authors' contributions

HMK and NMM put the study design and revised the manuscript. In addition, HMK was responsible for collecting and checking the clinical data of the studied patients and NMM revised the immunohistochemistry data. AAB: participated in the study design, carried out the immunohistochemistry and molecular studies and participated in drafing the manuscript. A-RNZ and MSM participated in performing the molecular studies and in drafting the manuscript. AMR participated in the immunohistochemistry and in revising the manuscript. All authors read and approved the final manuscript.

## Pre-publication history

The pre-publication history for this paper can be accessed here:

http://www.biomedcentral.com/1471-2407/9/32/prepub
